# CpG-set association assessment of lipid concentration changes and DNA methylation

**DOI:** 10.1186/s12919-018-0127-8

**Published:** 2018-09-17

**Authors:** Kaiqiong Zhao, Lai Jiang, Kathleen Klein, Celia M. T. Greenwood, Karim Oualkacha

**Affiliations:** 1 Department of Epidemiology, Biostatistics and Occupational Health, McGill University, 1020 Pine Avenue West, Montreal, Quebec, H3A 1A2 Canada; 2 Lady Davis Institute for Medical Research, Jewish General Hospital, 3755 Côte Ste. Catherine, Montreal, Quebec, H3T 1E2 Canada; 3 Department of Mathematics, Université du Québec à Montréal, 201, Ave. President Kennedy, Montreal, Montreal, H2X 3Y7 Canada; 4 Departments of Oncology and Human Genetics, McGill University, 3640 rue University, Montreal, Quebec, H3A 0C7 Canada

## Abstract

Epigenome association studies that test a large number of methylation sites suffer from stringent multiple-testing corrections. This study’s goals were to investigate region-based associations between DNA methylation sites and lipid-level changes in response to the treatment with fenofibrate in the GAW20 data and to investigate whether improvements in power could be obtained by taking into account correlations between DNA methylation at neighboring cytosine-phosphate-guanine (CpG) sites. To this end, we applied both a recently developed block-based data-dimension-reduction approach and a region-based variance-component (VC) linear mixed model to GAW20 data. We compared analyses of unrelated individuals with familial data. The region-based VC approach using unrelated (independent) individuals identified the gene *LGALS9C* as significantly associated with changes in triglycerides. However, univariate tests of individual CpG sites yielded no valid statistically significant results.

## Background

Lipid levels can be influenced by drug therapy or lifestyle factors such as diet, physical activity, alcohol consumption, and smoking [1]. Lipid levels are also associated with inherited genetic variants (single-nucleotide polymorphisms [SNPs]), as revealed by several genome-wide association studies [2]. However, DNA sequence variation explains only a small proportion of lipid-level variance [2]. Epigenetic modifications (eg, DNA methylation) alter DNA accessibility and hence can be involved in regulating patterns of gene expression. Through regulation of lipid levels, epigenetic mechanisms may contribute to cardiovascular risk profiles [3, 4]. Irvin et al. [3] identified strong association of 4 cytosine-phosphate-guanine (CpG) sites within the *CPT1A* gene on chromosome 11 with both triglycerides (TGs) and very-low-density lipoprotein C (VLDL-C). Because their analysis examined phenotype associations at each CpG site, a substantial correction for multiple testing was required.

This study’s goals were to investigate region-based associations between DNA methylation sites and lipid-level changes in response to the treatment with fenofibrate in the real data set provided by the GAW20 and to investigate whether improvements in power could be obtained by taking into account correlation between DNA methylation at neighboring CpG sites. We conducted 2 complementary block-based association tests that simultaneously accommodated all CpG sites that fell within a genomic region (block) for the purpose of investigating whether taking into account the correlation between DNA methylation at neighboring CpG sites and reducing the number of tests can improve power.

Turgeon et al. [5] have proposed “principal components of explained variation (PCEV).” The PCEV approach integrates, simultaneously, an optimal data-dimension-reduction technique with testing for association. It provides analytical and empirical *p* value calculations for testing association between a set of correlated variables (eg, methylation profiles of a genomic region) and 1or more variables of interest (eg, high-density lipoprotein [HDL]/TG).

We contrast PCEV with a variance components (VC) score test method. This is a sequence kernel association test (SKAT)-type association test that decomposes the total variance of a phenotype (eg, HDL/TG) into the variance explained by a block/region-methylation profiles and a residual variance term [6]. Specifically, the model assumes that the phenotypic similarity between subjects is captured by the region–methylation similarity. The VC-score approach significantly reduces the model degrees of freedom compared to standard multivariate regression models.

## Methods

Suppose we have observed data {***Y***, ***x***, ***C***} where ***Y*** is an *n* × *p* matrix of *n* subjects for which a block of *p* variables/phenotypes are measured (eg, methylation values at a genomic region/gene with *p*CpG dinucleotides), ***x*** is an *n* × 1 vector of an observed trait of interest (eg, HDL phenotype) and ***C*** is an *n* × *r* matrix where the columns are known confounding factors (eg, age, sex).

### PCEV approach

PCEV is a dimension-reduction technique that searches for a linear combination (a principal component) of the columns of ***Y***, ***y***_*pcev*_ = ***Y w*** (***w*** is a*p* × 1 vector), that maximizes *h*^2^(***w***), the ratio of the variance in ***Y*** explained by ***x*** to the total variance of ***Y***, while taking into account the confounding factors, ***C***. This new score ***y***_*pcev*_ can then be used as a phenotype in standard statistical models to test for the relationship between ***Y*** and ***x***. Searching for ***y***_*pcev*_ is equivalent to projecting the rows of ***Y*** into ***w***, where ***w*** is the most relevant direction in p-dimension space to ***x***. A linear relationship between ***Y*** and ***x*** can be tested by ***H***_01_ : *corr*(*y*_***pcev***_, *x*) = 0. This test requires the use of the data twice, and therefore a naïve approach for *p* value calculation will suffer from Type I error inflation. However, the null ***H***_01_ is equivalent to testing for ***H***_02_ : *h*^2^(***w***_***pcev***_) = 0 which uses the data only once. Turgeon et al. [5] derived an analytic test for the null hypothesis ***H***_02_, which was shown to yield the proper Type I error rate.

### VC-score approach

In a reverse model where ***x*** (eg, HDL) is modeled as the response variable and ***Y*** as a design matrix of *p* predictors, the VC model links ***x*** to ***Y*** using a linear mixed-effects regression model in which ***Y*** has an effect on the variance of ***x*** instead of on its mean [6]. This approach was developed to test association between a set of rare variants and a phenotype of interest. However, the test can be adapted easily to handle different types of design matrices, such as methylation from a genomic region of interest. This method can be extended to take into account population and family structures. The family-based VC-score approach is a linear mixed-effects model in which a second random effect for genetic relationships (ie, kinship) is added [7].

### Phenotypic, methylation, and covariate data

Circulating blood lipids, HDL, TGs, and the methylation profiles were measured at baseline and following 3 weeks of daily treatment with 160 mg of micronized fenofibrate [2]. For this study, we investigated HDL and TG changes among 714 participants for whom pretreatment methylation data were available. Because the PCEV approach has only been implemented for use with independent subjects, analyses using this method were conducted using 242 unrelated individuals. The selection of the maximum set of unrelated individuals from each pedigree was done using a greedy algorithm that used the kinship matrix to sequentially remove related individuals [8]. Log-transformations were performed for TG, as this variable was not normally distributed.

T-cell pre- and posttreatment DNA-methylation at 463,995CpG sites were already normalized using *ComBat* [3]. These CpG sites were allocated to 22,319 genes. We also included sites located 20 kb up- and downstream of the gene region. Only the CpG sites with gene annotations were evaluated in the analyses; consequently, we analyzed 401,326 CpG sites. Because PCEV works when the block and sample sizes are comparable [5], we divided the largest gene blocks to obtain 22,488 gene regions with no more than 130 CpG sites per block.

We focused on the pretreatment methylation levels to evaluate the effect of individual CpG sites and genes on explaining the observed heterogeneity in response to treatment. To capture unwanted variability in methylation profiles, which could result from variation in cell purity or batch effects, we constructed principal components of genome-wide methylation levels using 2000 randomly sampled probes from all autosomes. The association analyses between pretreatment methylation probes and blood lipid changes were adjusted for age, sex, study center, smoking status, diagnosed metabolic syndrome status, the fast time on the pre- and posttreatment visits, and the top 4 methylation-derived principal components (PCs).

## Results

Bonferroni thresholds for significance at a 10% family-wise error rate were established using 401,326 univariate tests and 22,488 CpGset tests. No univariate tests for TG changes passed this threshold. The family-based VC-score approach identified 2 genes (*RNMT* and*MIR130B*) as significantly associated with HDL changes. The VC-score approach using unrelated subjects identified the gene *LGALS9C* as significantly associated with TG changes. Table 1 lists the 5 most significant genes with association to lipid changes identified by univariate, PCEV and VC-score approaches. Among the top 5 genes, both region-based approaches identified *NUDCD3* for its relationship with HDL changes among independent participants. There is no overlap among the top 5 genes for TG changes identified by the 3 approaches.

**Table 1:** Top 5 individual CpG sites and the top 5 genes identified by VC-score and PCEV approaches that are associated with HDL and TG changes, using unrelated and familial individuals

Unrelated individuals (sample size = 242)										Families (sample size = 714)						
Univariate				VC-score			PCEV			Univariate				VC-score		
CpG	Genes	Chr	*p* Value	Genes	Chr	*p* Value	Genes	Chr	*p* Value	CpG	Genes	Chr	*p* Value	Genes	Chr	*p* Value
HDL																
cg14258154	*ZFYVE9*	1	1.56E-06	*ALDH1A2*	15	2.85E-05	***NUDCD3***	7	1.87E-04	**cg02273903**	*KIAA1199*	15	3.30E-08*	***RNMT*** ^†^	**18**	2.55E-06*
cg26385523	*CTU1*	19	3.32E-06	*CSF1R*	5	5.18E-05	*LIN28B*	6	2.01E-04	**cg21250577**	*ETFA*	15	5.24E-08*	***MIR130B***	**22**	3.09E-06*
cg00638075	*RNF40†*	16	4.04E-06	***NUDCD3***	7	5.57E-05	*NID1*	1	7.68E-04	**cg00979026**	*LAMP1*	13	8.52E-08*	*C6orf141*	6	5.38E-06
cg27639620	*TSPAN4*	11	6.36E-06	*ACD*	16	1.17E-04	*CSMD1*	8	1.10E-03	**cg21654314**	*CHMP6*	17	1.40E-07*	*TUBB3*	16	7.03E-06
cg26671183	*PFKFB2*	1	6.48E-06	*C17orf57*	17	1.51E-04	*ECEL1P2*	2	1.24E-03	**cg17990398**	*JAKMIP3*	10	1.60E-07*	*TBX15*	1	9.01E-06
TG																
cg18522239	*LGALS9C*	17	2.04E-06	***LGALS9C***	**17**	**3.12E-06** ^*^	*PTPRN*	7	1.58E-04	cg12110750	TSHZ3	19	3.09E-06	*MIR941–1* ^†^	20	3.07E-05
cg05984096	*CSK*	15	7.63E-06	*ZNF592*	15	1.20E-05	*RSPO2*	8	3.73E-04	cg02565993	FIBIN	11	3.50E-06	*TP53I11*	11	4.07E-05
cg06043820	*ZNF592*	15	9.86E-06	*HIPK2*	7	1.19E-04	*FAM86A*	16	5.66E-04	cg08552519	PCBP4	3	6.02E-06	*GKN1*	2	4.60E-05
cg04902851	*NSMAF*	8	1.18E-05	*SPAG8*	9	1.83E-04	*HOXD13*	2	8.30E-04	cg04004830	SDPR	2	6.15E-06	*PRR4* ^*†*^	12	5.09E-05
cg10243301	*ZNF682*	19	1.43E-05	*CSK*	15	3.03E-04	*C14orf28*	14	1.24E-03	cg09969806	KIF1A	2	6.43E-06	*LOC100216545*	7	6.61E-05

*Significant at 10% family-wise error rate.

^†^ *RNMT:RNMT;C18orf19. MIR941–1: MIR941–1; DNAJC5. PRR4: PRR4; PRH1; TAS2R43. RNF40: RNF40;C16orf93.*

**Fig. 1:**
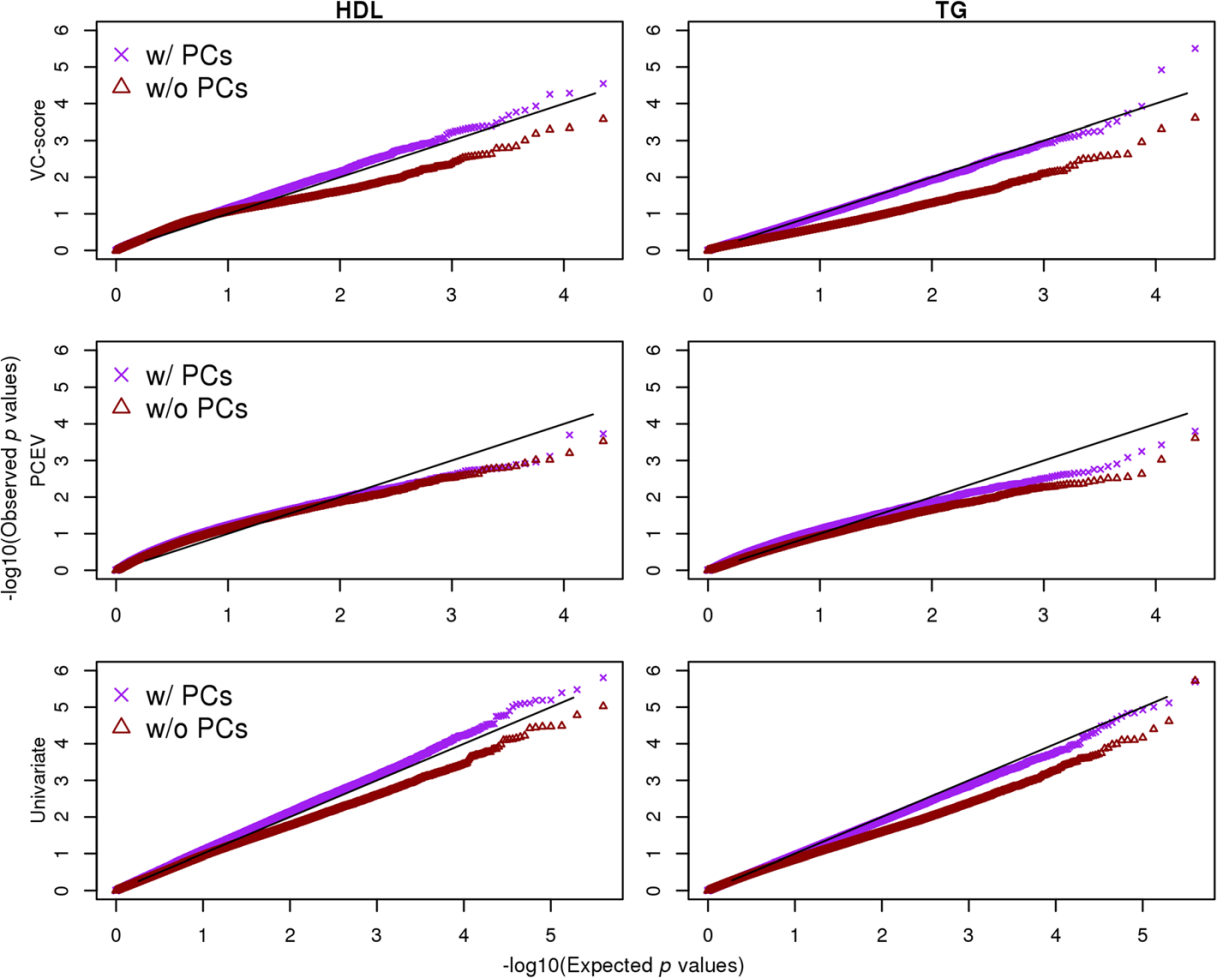
Q-Q plots for gene-based *p* values obtained from VC-score (*top*), PCEV (*middle*), and the individual CpG-based *p* values (*bottom*) for the HDL (*left*) and TG (*right*) changes, using the 242 independent participants The results without the extra adjustment of 4 methylation principal components are also shown for contrast.

**Fig. 2:**
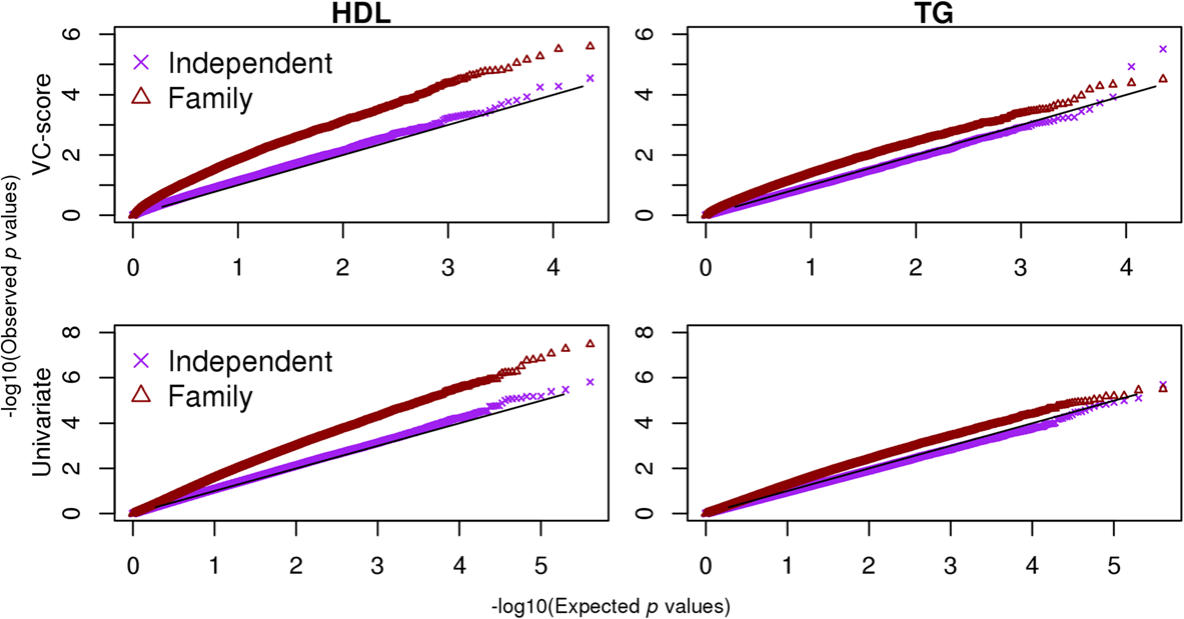
The Q-Q plots for the gene-based (VC-score [*top*]) and individual (*bottom*) CpG-based *p* values comparing analysis of just the independent participants with families. All analyses were adjusted for the top 4 methylation-derived PCs

Figure 1 shows quantile–quantile (Q-Q) plots for the gene-based *p* values and the individual CpG-based *p* values for the HDL changes and TG changes, using data from the 242 unrelated individuals. Under each analysis, adjustments with and without the 4 methylation PCs were compared, revealing that inclusion of these PCs was important in controlling for unknown confounding. Without this extra adjustment, the distribution of *p* values was very biased away from what would be expected under the null. Figure 2 contrasts the results obtained using the independent participants and the ones using the families. Inclusion of all family participants can increase power, but also inflated the *p* values compared to the null.

## Discussion

In this study, we investigated associations between DNA methylation and lipid-level changes in response to fenofibrate treatment in the GAW20 real data set. The smallest *p* value found by both the VC-score test for TG differences and the single-CpG association test was at gene *LGALS9C*. However, as a result of multiple-testing power-loss issues, no single-CpG test passed a Bonferroni-corrected threshold. Simulation studies are necessary for more rigorous power assessments comparing region-based and univariate methods. Furthermore, we did not replicate the results of Irvin et al. [3] for baseline lipids, although this is not surprising as we analyzed lipid-level changes. We focused on linear relationships; however, nonlinear association methods may be more favorable/powerful when the primary interest is lipid-level changes.

In all analyses, we adjusted for available/known confounders and for unknown confounders using 4 PCs calculated based on 2000 CpG probes selected randomly from available DNA methylation on all chromosomes. We also contrasted this with an analysis using PCs calculated from all CpG sites, and found little difference in the results (not shown). The adjustment resulting from the PCs improved the validity of VC-score test results; however, (unusual) deviation of the PCEV test statistic from the null distribution was also indicated. This might be a consequence of the nonrobustness of PCEV to violation of normality assumption or the constant variance assumption of model residuals (errors). Even after normalization, methylation proportions have variances that are small when means are near 0 and 1. This heteroscedasticity might lead to a loss of power [9]. Thus, a transformation such as the *logit* may help in obtaining test statistics with valid null distributions.

We considered 2 ways to accommodate the relatedness among the participants: to restrict the analysis to unrelated individuals or to use a linear mixed model that takes family structure into account. In contrast to the well-behaved *p* value distribution for the analysis of unrelated subjects, the family-based VC-score test showed inflation in the Q-Q plots. Hence, the significant results for the 2 genes *RNMT* and *MIR130B* may be questionable. Almeida et al. [10] found that heritability of pretreatment DNA methylation was much higher than expected. Our results agree that pedigree-based kinship corrections are insufficient to correct for familial correlations in DNA methylation, and that additional corrections must be considered.

Other region-based association methods may be worth exploring in the future, such as the global analysis of methylation profiles (GAMP) [11] in which the density of methylation values in a region is approximated by B-splines and then the spline coefficients for each individual are used as covariates in association tests. Other strategies to accommodate the family structure in the region-based association tests include MF-KM (multivariate family data using kernel machine regression) [12], a linear mixed model built upon kernel machine regression, and mFARVAT (multivariate family-based rare variant association tool) [13], a quasi-likelihood-based-score test approach.

## Conclusions

The region-based VC approach using unrelated individuals identified the gene *LGALS9C* as significantly associated with changes in triglycerides. However, univariate tests of individual CpG sites yielded no valid statistically significant results. After correctly accounting for the unknown confounding and subject relatedness, region-based methods show an improvement in power to detect associated genes as compared to single-marker methods.
